# Knowledge of Saudi dental students and interns towards luting cements and their applications in fixed prosthodontics

**DOI:** 10.1186/s12903-023-03054-3

**Published:** 2023-05-29

**Authors:** Passent Ellakany, Reem Abualsaud, Mohammed M. Gad, Sara Atteya, Omar A. El Meligy, Osama A. Qutub, Amr A. Mahrous

**Affiliations:** 1 Department of Substitutive Dental Sciences, College of Dentistry, Imam Abdulrahman Bin Faisal University, Dammam, Saudi Arabia; 2 Department of Pediatric Dentistry and Dental Public Health, Faculty of Dentistry, Alexandria University, Champollion St, Azarita, 21527 Alexandria Egypt; 3 Department of Pediatric Dentistry, Faculty of Dentistry, King Abdulaziz University, Jeddah, Saudi Arabia; 4 Department of Oral and Maxillofacial Prosthodontics, Faculty of Dentistry, King Abdulaziz University, Jeddah, Saudi Arabia

**Keywords:** Knowledge, Fixed dental prosthesis, Dental cement, Prosthodontics, Dental students, Dental interns

## Abstract

**Background:**

Enhancement of students’ knowledge is essential in improving their clinical skills and performance. Thus, the curriculum should be prepared to achieve a better outcome. The current study aimed to determine the dental students’ and interns’ basic knowledge towards dental luting cements and their application in dental practice to improve the theoretical and clinical training sections.

**Materials and methods:**

A cross–sectional study was conducted among dental students and interns at three Colleges of Dentistry in the Kingdom of Saudi Arabia between September 2019–June 2020. An online questionnaire was used which included demographic data, questions about luting cement usage, cementation techniques, and commonly used cements in dental clinics. Descriptive analysis and chi–square test were used to show the association between level of dental education and the use of dental cements using SPSS software. The significance level was set at 5%.

**Results:**

The total respondents were 626 dental students/interns of whom 78.8% were undergraduate dental students. Participants who reported undergraduate studies as the source of information were 79.7%. The type of restoration was the main factor in luting cement selection (62.6%). Concerning the isolation technique in cementing laminate veneers, 49.7% used dri–angles, cotton rolls and saliva ejectors. Dual–cure resin cement was the most common cement used in all the mentioned restorations except in pressed porcelain laminate veneers and cement–retained implant–supported restorations.

**Conclusions:**

Students’ knowledge and practice in managing dental implants and porcelain laminate veneers need to be improved. The selection of a luting agent for a given restoration by students and interns was based on the basic knowledge, available cement, and the type of restoration. Awareness towards the management of short prepared teeth and custom-made cast posts and cores is also limited.

**Supplementary Information:**

The online version contains supplementary material available at 10.1186/s12903-023-03054-3.

## Background

The major function of dental cement is sealing the gap between the prepared tooth and restoration and retaining the restoration in place [1]. Old dental cements were used mainly to retain restorations mechanically but the latest cements depend on chemical adhesion besides mechanical bonding, that’s why the synonym crown cement is widely used nowadays [2–5]. Optimum dental cement should be biocompatible, have adequate working time, short setting time, low solubility, superior bond and compressive strengths, be esthetic, easy to manipulate and clean–up, and affordable [4–6].

Dental cements are classified into liners and bases, permanent or provisional according to their durability [4, 7]. Provisional cements include calcium hydroxide, zinc oxide eugenol (ZOE) or eugenol–free cement (ZONE). Previously, ZOE cement was used due to its obtunding effect on the pulp. Later, it was discovered that the eugenol inhibits the polymerization of composite resin, that’s why non–eugenol temporary cements became more preferred [8, 9].

Cements like zinc phosphate (ZP), zinc polycarboxylate (ZPC), conventional glass–ionomer (GIC), resin–modified glass–ionomer (RMGIC), and resin cement (RC) are considered permanent cements used in the final cementation procedure [7, 10, 11]. ZP is the gold standard cement although it exhibits only physical bond to tooth structure, high solubility and post–cementation hypersensitivity [10, 11]. However, ZPC exhibits chemical adhesion to tooth structure with a weak bond to enamel and dentin [11]. GIC exhibits anticariogenic properties through long–term leach out of fluoride but has a weak bond, especially in case of excessive dehydration resulting in post–cementation sensitivity [5, 11]. RMGIC intermingles the properties of dental RC and conventional GIC [12]. RC exhibits high strength, adhesive bond, low solubility as well as anticariogenic and high esthetic properties [11]. RCs are available in different polymerizing modes such as light–, self– or dual–cure cements [13, 14]

Up to our knowledge, the literature is deficient in studies reporting the level of basic knowledge of students/interns in the Kingdom of Saudi Arabia (KSA) population regarding the type of cements, cementation techniques and cement applications. The current study aimed to assess the basic knowledge of dental students and interns in KSA, to determine the adequacy of the taught courses, and to determine the students/interns information about techniques, types, and indications of cements used in different clinical situations. This would provide comprehensive information about the areas that need to be added or elaborated in the curriculum of dental prosthodontic courses throughout the dental educational years; thus, the students’ and interns’ theoretical and clinical knowledge might be enhanced. Based on this framework, the research questions were: (i) what were the sources of information used by dental students and interns to get information about luting cements? and (ii) were the clinical courses given to dental students during their undergraduate studies competent in providing the correct information regarding type, uses, precautions, and indications?

## Methods

### Design

A cross–sectional study was conducted among dental students/interns practicing clinical procedures that involve the usage of dental cements. Participants were invited from three Colleges of Dentistry in KSA: Imam Abdulrahman Bin Faisal University (IAU) in Dammam, King Saud University (KSU) in Riyadh, and King Abdulaziz University (KAU) in Jeddah between September 2019–June 2020. Ethical approval was obtained from the Research Ethics Committee at Imam Abdulrahman Bin Faisal University (E.A. 2,017,029).

### Participants and sample size

A proper sample size of representative dental students/interns was involved in the questionnaire. Exclusion criteria included students involved in preclinical years. The sampling strategy relied on seeking responses from the largest number of registered dental students/interns. The sample was available dental students in clinical years and dental interns from three Colleges of Dentistry in KSA: IAU, KSU, and KAU about 860 participants.

### Questionnaire design and pilot testing

An online survey was developed using the survey monkey tool (www.surveymonkey.com). The survey link was distributed over the university email lists of eligible students (registered in clinical dental years) and interns. A reminder email was sent to the students/interns after two weeks. The questionnaire was prepared to assess the awareness of a sample of dental students and interns in the KSA per level towards basic knowledge of luting cements and the competency of clinical dental courses in these dental colleges. A brief introduction was added to the questionnaire including the objectives of the study, the duration required to answer the questionnaire, and confirming anonymous and voluntary participation. Each participant could submit only one online questionnaire where all questions were required to be answered.

### Content and face validity of the questionnaire

The content validity form was distributed on four dental experts to rate the degree of relevance of each question on a four–point ordinal scale: (1) not relevant, (2) somewhat relevant, (3) fairly relevant and (4) highly relevant. We computed the content validity at the item level (CVI–I) by dividing the total number of experts by the number of those who gave a score of 3 to 4 for each item. The overall CVI–I score was 0.95 which was considered appropriate [15].

A pilot study was conducted to assess the duration needed to fill the survey and overcome any difficulties in the comprehension of the questions. A group of experts evaluated the English language of the questionnaire and confirmed the validity and logical structure of the questions. The questionnaire was further tested among 20 dental students and dentists at different stages of their careers to examine its face validity by using a dichotomous scale with “Yes” and “No” options denoting clear and unclear item, respectively [16], and few modifications for clarification were done. Cohen’s Kappa test of the responses was calculated yielding a score of 0.87, which was considered a good agreement.

The questionnaire was divided into three main sections (Appendix I). The first section included personal and professional information (age, gender, level of dental education (students/interns), affiliation, and percent of patients requiring the use of luting cement). The second section consisted of four questions about practices related to the usage of luting cement such as the source of knowledge, factors to consider when selecting the type of cement, management of gingival bleeding, and isolation technique in laminate veneers cementation. The third section comprised matching eight clinical situations with the suitable cement (ten different types).

### Statistical analysis

All data were presented as simple counts and percentages. The significance level was set at 5%. Descriptive analysis and chi–square test were done to show the association between the level of dental education and the use of dental cements using SPSS (IBM SPSS Statistics for Windows, Version 27.0. Armonk, NY, IBM Corp. 2020). Charts were formatted for an easy depiction of the results. Multiple response analysis was done since each question had more than one option to choose from.

## Results

Responses were obtained from 626 dental students and interns with a 72.3% response rate. The responses were 400/470 (85.1%), 103/200 (51.5%) and 123/190 (64.7%) participants from IAU, KSU, and KAU, respectively. Of these, 52.6% were males and 78.8% were undergraduate dental students. About 43% used cements for < 25% of the cases and 31.6% used dental cements for 25–50% of the patients (Table 1). There was a statistically significant difference between the level of dental education (undergraduate students vs. interns) and the percentage of patients requiring the use of dental cement (*P* < 0.001), with the majority being dental interns (93%) (Table 2).

**Table 1:** Personal and demographic data

Factor		Number (Percentage)
**Gender**	Male	329 (52.6)
	Female	297 (47.4)
**Participant**	Undergraduate Students	493 (78.8)
	Interns	133 (22.2)
**What percentage of your patients requires the use of luting cements?**	0	70 (11.2)
	1–25%	269 (43)
	> 25%–<50%	198 (31.6)
	> 50–<75%	51 (8.1)
	> 75%	38 (6.1)
**Total**	626	

**Table 2:** The association between the level of education (undergraduate students vs. interns) and the percentage of patients that requires use of luting cement

Participant	Percentage of patients requiring cementation					Total
	0	1–25%	> 25%–<50%	> 50–<75%	> 75%	
**Undergraduate Students N (%)**	66 (13.4)	212 (43)	140 (28.4)	45 (9.1)	30 (6.1)	493
**Interns N (%)**	4 (3)	57 (42.9)	58 (43.6)	6 (4.5)	8 (6)	133
Test of sig* ( ***P*** **–value)**	20.496 (< 0.001)					

* Chi–square

**Fig. 1:**
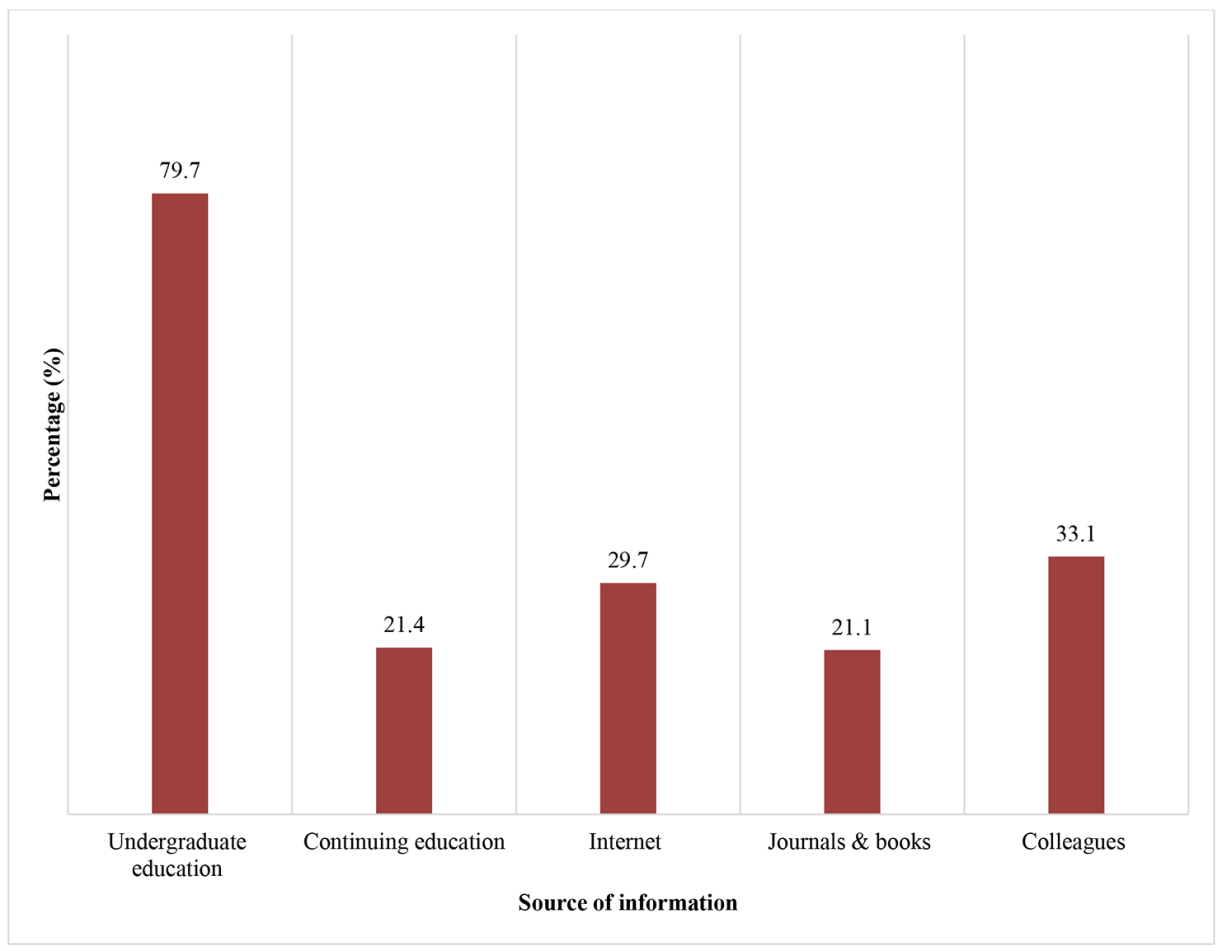
Sources of information about luting cements * In case of traumatic bleeding ** In case of bleeding from inflammation

**Fig. 2:**
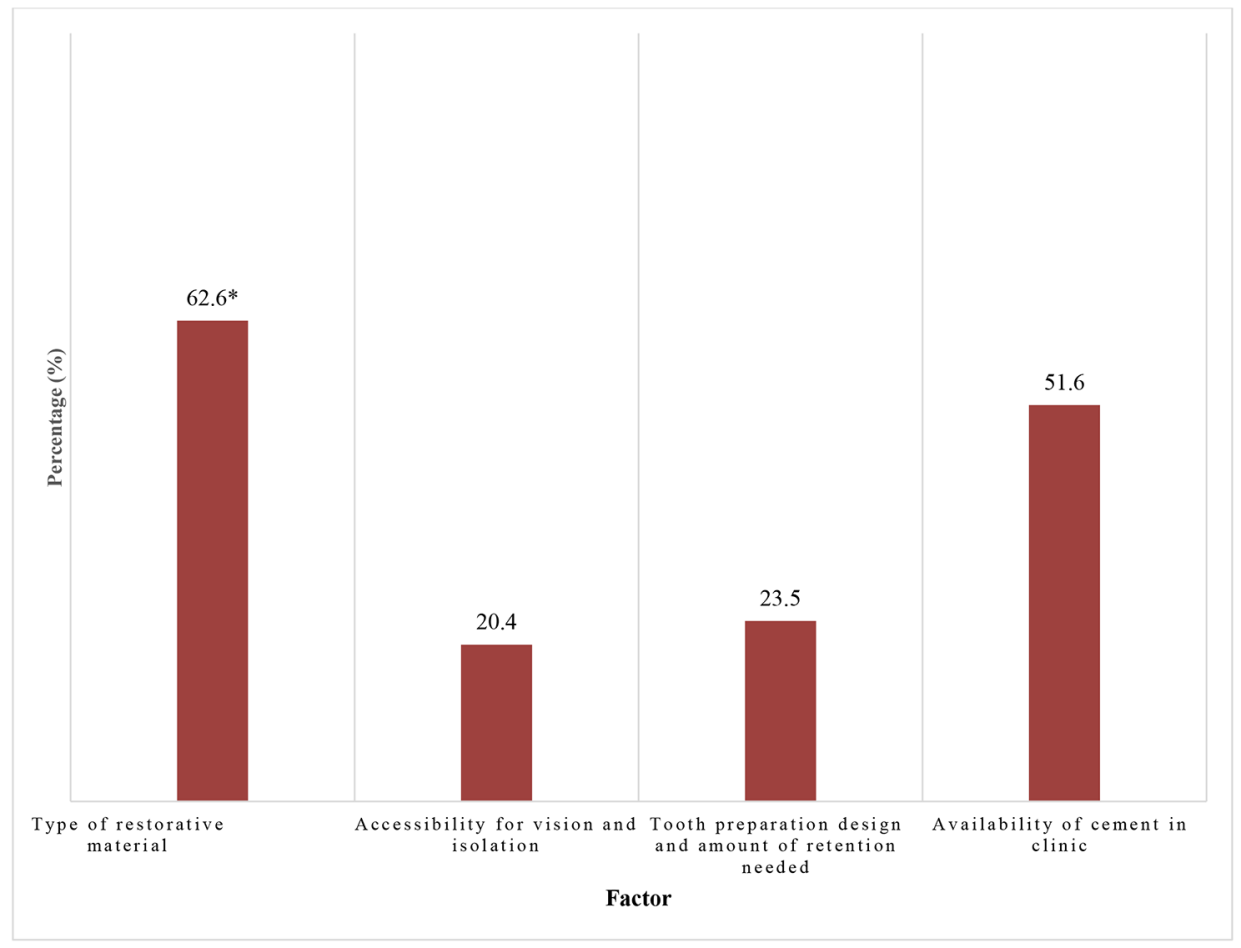
The factor affecting the selection of luting cement * The most important factor in selecting the optimum cement

**Fig. 3:**
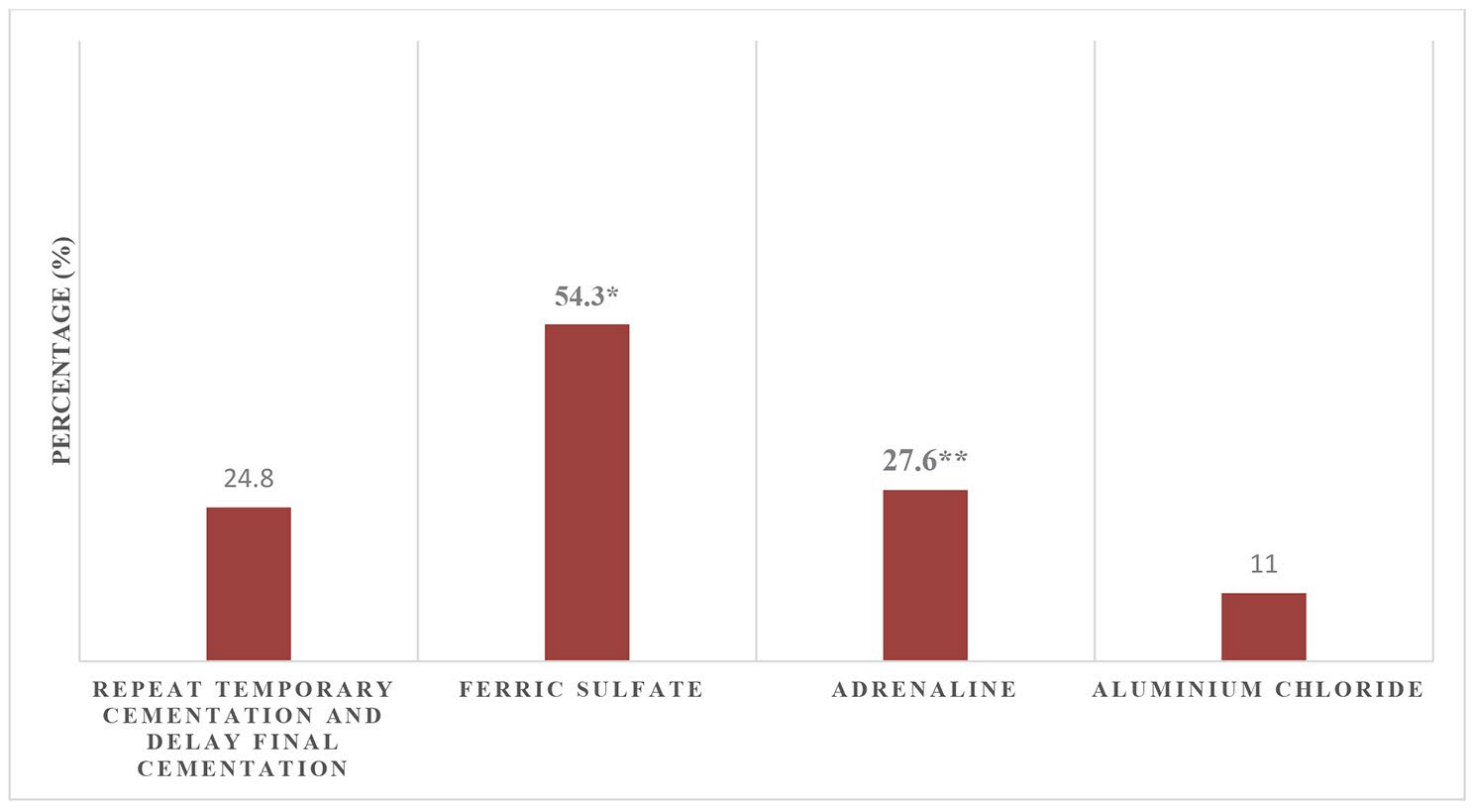
**Techniques** to deal with gingival bleeding during final cementation

The majority of participants (79.7%) got their information about cements from undergraduate education (Fig. 1). The most important factor in selecting the optimum cement was the type of restoration (62.6%) (Fig. 2). As for the technique used to deal with gingival bleeding during final cementation, 54.3% preferred applying ferric sulfate hemostatic agent, while 27.6% and 24.8% used adrenaline or repeated temporary cementation, respectively (Fig. 3). Concerning the isolation technique used for cementing laminate veneers, 49.7% used dri–angles, cotton rolls and saliva ejectors and 37.7% used retraction cords.

Table 3 shows the luting cements used in different prosthodontic cases. In case of a short–prepared tooth, (17.8%) of undergraduates and (15%) of interns used GIC, while (15.5%) and (11.3%) used its modified formed (RMGIC), respectively. Provisional restorations were mainly cemented using ZOE (by (50.4%) of interns and (27.8%) of undergraduates and ZONE by (29.4% and 19.5%) respectively. Glass fiber posts were commonly cemented using dual–cure RC were (42.6%) and (43.6%) for undergraduates and interns, respectively, while custom–made cast post and cores were cemented by undergraduates using GIC (46.6%), and dual–cure RC by interns (29.3%). Lithium disilicate restorations were cemented using dual–cure (21.5%) by undergraduates and light–cure RC (24.1%) by interns. For zirconia–based restorations, (17.3%) of interns used GIC and (16.4%) of undergraduates used dual–cure RC for cementation, while pressed porcelain laminate veneers were cemented using light–cured and dual–cure RC (18.8% of interns and (15.8%) of undergraduates), respectively. Finally, cement–retained implant–supported restorations (CRIS) were cemented using GIC (8.3%) of interns, chemical cure resin (7.9% of undergraduates), and dual–cure resin (7.7%). Regarding the applicability of these types of restorations, the least made prosthesis by participants was the CRIS (55.6%), followed by pressed porcelain laminate veneers (45.7%), zirconia–based restorations (38.2%), and lithium disilicate restorations (37.7%), while the prefabricated glass fiber post was the most commonly made restoration (86.3%).

**Table 3:** Luting cement use in different prosthodontic cases

Cases		Zinc oxide eugenol (ZOE)	Zinc oxide non–eugenol (ZONE)	Zinc phosphate (ZP)	Zinc poly–carboxlate (ZPC)	Glass ionomer (GIC)	Resin modified glass ionomer (RMGIC)	Chemical–cure resin	Dual–cure resin	Light–cure resin	I don’t do this type of restorations / or none of the above
1. **Short prepared tooth**	Undergraduate	48 (9.7)	20 (4.1)	20 (4.1)	28 (5.7)	88 (17.8)	82 (15.5)	25 (5.1)	55 (11.2)	13 (2.6)*	114 (23.6)
	Interns	8 (6)	2 (1.5)	3 (2.3)	1 (0.8)	20 (15)	15 (11.3)	1 (0.8)	21 (15.8)	3 (2.3)	59 (44.4)
2. **Provisional acrylic restoration**	Undergraduate	137 (27.8)*	145 (29.4)*	33 (6.7)	16 (3.2)*	38 (7.7)	4 (0.8)	24 (4.9)	10 (2)	5 (1)	81 (16.4)
	Interns	67 (50.4)*	26 (19.5)*	3 (2.3)	4 (3)*	16 (12)	6 (4.5)	3 (2.3)	2 (1.5)	0	6 (4.5)
3. **Prefabricated glass fiber post**	Undergraduate	10 (2)	6 (1.2)	18 (3.7)	10 (2)	33 (6.7)	50 (10.1)	40 (8.1)*	210 (42.6)*	32 (6.5)	82 (16.6)
	Interns	5 (3.8)	4 (3)	2 (1.5)	2 (1.5)	27 (20.3)	14 (10.5)	3 (2.3)*	58 (43.6)*	14 (10.5)	4 (3)
4. **Custom–made cast post & core**	Undergraduate	11 (2.2)	1 (0.2)	44 (8.9)*	25 (5.1)	60 (12.2)*	50 (10.1)	38 (7.7)	91 (18.5)	19 (3.9)	151 ( 30.6)
	Interns	1 (0.8)	2 (1.5)	0	4 (3)	62 (46.6)*	10 (7.5)	2 (1.5)	39 (29.3)	0	13 (9.8)
5. **Lithium disilicate restorations**	Undergraduate	11 (2.2)	4 (0.8)	10 (2)	16 (3.2)	39 (7.9)	65 (13.2)	17 (3.4)	106 (21.5)*	47 (9.5))*	178( 36.1)
	Interns	2 (1.5)	0	0	0	11 (8.3)	4 (3)	8 (6)	18 (13.5)*	32 (24.1)*	58 (43.6)
6. **Zirconia based restorations**	Undergraduate	16 (3.2)	9 (1.8)	24 (4.9)	16 (3.2)	62 (12.6)*	45 (9.1)*	11 (2.2)	81 (16.4)	40 (8.1)	189 (38.3)
	Interns	1 (0.8)	4 (3)	3 (2.3)	2 (1.5)	23 (17.3)*	13 (9.8)*	1 (0.8)	21 (15.8)	15 (11.3)	50 (37.6)
7. **Pressed porcelain laminate veneers**	Undergraduate	5 (1)	10 (2)	21 (4.3)	13 (2.6)	32 (6.5)	28 (5.7)	27 (5.5)	70 (14.2)	69 (14)*	216 (43.8)
	Interns	1 (0.8)	0	2 (1.5)	2 (1.5)	6 (4.5)	2 (1.5)	5 (3.8)	21 (15.8)	25 (18.8)*	69 (51.9)
8. **Cement retained implant supported restorations**	Undergraduate	21 (4.3)	11 (2.2)*	21 (4.3)	24 (4.9)	39 (7.9)*	33 (6.7)	39 (7.9)	38 (7.7)	12 (2.4)	253 (51.3)
	Interns	3 (2.3)	3 (2.3)*	2 (1.5)	1 (0.8)	11 (8.3)*	5 (3.8)	5 (3.8)	7 (5.3)	1 (0.8)	95 (71.4)

* The most suitable types of dental cements in relation to the restoration used according to literature

No statistical significance was found between the level of dental education (dental students versus interns) and the undergraduate education as a source of information about luting cements nor the type of restoration as the most important factor when choosing type of luting cement (Tables 4 and 5).

**Table 4:** Association between the level of education (students vs. interns) and the undergraduate education as a source of information about luting cements

Participant	Undergraduate education as a source of information about luting cements		Total
	Yes	No	
**Undergraduate students N (%)**	401(81.34%)	92 (18.66%)	493
**Interns N (%)**	98 (73.68%)	35 (26.32%)	133
**Total**	499	127	626
Test of sig* ( ***P*** **-value)**	3.7949 (0.51407)		

* Chi-square

**Table 5:** Association between the level of education (students vs. interns) and the type of restoration as the most important factor when choosing type of luting cement

Participant	Type of restoration as the most important factor when choosing type of luting cement		Total
	Yes	No	
**Undergraduate students N (%)**	316 (64.1%)	177 (35.9%)	493
**Interns N (%)**	76 (57.14%)	57 (42.86%)	133
**Total**	392	234	626
Test of sig* ( ***P*** **-value)**	2.164 (0.141255)		

* Chi-square

Regarding management of bleeding during cementation, there was a statistically significant difference between students and interns in managing bleeding during cementation in all methods except ferric sulfate (Table 6).

**Table 6:** Association between the level of education (students vs. interns) and management of bleeding during cementation

Participant	Management of bleeding during cementation		Total	Test of sig* (*P*-value)
	Repeat temporary cementation and delay final cementation			
	Yes	No		
**Undergraduate students N (%)**	111 (22.5%)	382 (77.5%)	493	0.017**
**Interns N (%)**	44 (33.1%)	89 (66.9%)	133	
**Ferric sulfate**				
**Undergraduate students N (%)**	269 (54.6%)	224 (45.4%)	493	0.845
**Interns N (%)**	71 (53.4%)	62 (46.6%)	133	
**Adrenaline**				
**Undergraduate students N (%)**	147 (29.8%)	346 (70.2%)	493	0.021**
**Interns N (%)**	26 (19.5%)	107 (80.5%)	133	
**Aluminum chloride**				
**Undergraduate students N (%)**	65 (13.2%)	428 (86.8%)	493	< 0.001**
**Interns N (%)**	4 (3%)	129 (97%)	133	

* Chi-square

** Statistical significant

## Discussion

The current study reported aspects related to the use of dental cements, unlike other studies that evaluated the dentists’ knowledge of resin–bonded prosthesis which is not commonly practiced by all dentists [17, 18]. The results of this survey revealed that participants selected the cement depending on the restorative material to be cemented (62.6%). This agrees with the findings of Lad et al [5] who reported that the proper selection of dental luting cement relies mainly on the restorative material.

Regarding the number of patients requiring cementation of dental restorations by participants, about 43% of participants used dental cements for 1–25% patients/week, while 31.6% used dental cements for > 25%–<50% patients/week. This is supported by the distribution of the participants in this survey, with the majority being undergraduate students and only 21.2% being interns. Undergraduate students have didactic sessions besides clinical courses which limit their clinical time and therefore the number of patients seen every week. This might be justified by the larger number of participants seeing a lower number of patients requiring cementation of indirect restorations. To add to that, students were consuming more time in the clinical session consulting their supervisors to approve each clinical step unlike interns having more freedom in performing the treatment independently.

The quality of dental treatment depends mainly on the dentist’s knowledge and clinical skills. Those two factors are reliant on the dentist’s educational background and training [19]. The results of this survey showed that the majority of participants depended on their undergraduate education (97.7%) to select the appropriate cement for each situation. Following that was the information shared by colleagues (33.1%). The increase in social media access and internet searches these days [20, 21] supported the high percentage of students reaching out to the internet for information (29.7%). A smaller percentage of the participants still referred back to reference books and journals (21.1%).

Since this study surveys the practice of dental students and interns within academic institutions, it is understood that some limitations to the freedom of cement selection may apply. Around one–fifth of the participants selected cements based on the degree of accessibility and possibility to isolate the operative field [1]. This might be linked to the type of cement used and the need of strict moisture control during setting/polymerization to avoid contamination and drop in cement properties [1, 22]. Also, whether rubber dam isolation is clinically achievable or not, as well as the accessibility to remove excess cement, especially in the posterior region where access is difficult, embrasure is smaller and proximal contacts are broader [4, 5].

In the present study, 54.3% of the participants supported the use of ferric sulfate to control the bleeding during cementation. Saliva and water must also be controlled during cementation to ensure proper visibility and cement mechanical properties. For laminate veneer cementation, half the participants reported the use of cotton rolls, dri–angles and saliva ejectors followed by the use of retraction cords (37.7%). The use of split section rubber dam was reported by (17.4%) and only (9.9%) used individual tooth rubber dam isolation. These results were in accordance with those reported by Pavithra [23], who stated the use of cotton rolls and retraction cords to account for 85%, and 11.3%, respectively. On the other hand, Naram and Pradeep [24] reported that half the students used rubber dams for isolation during cementation. However, their study focused on cementation of resin–bonded restorations.

With regards to luting agents used for the cementation of fixed restorations, there was a great variation in the responses concerning the type of cement per restoration type. In some cases, the restorative dentist is faced with a short preparation that cannot be lengthened surgically or non–surgically. Therefore, there will be great reliance on the retentive ability of the cement used to retain that restoration. The recommendation is to use cement that has high tensile and compressive strengths with low solubility such as RC [25, 26]. In this study, the participants selected RC (18.9%), GIC (17.35) and RMGIC (15.5%). Lawson et al. [1] reported that short and excessively prepared abutments need to be bonded adhesively which supports the results of the current study.

The results of the present study revealed that 59.9% used ZO cement for provisional restoration cementation. Out of which 45.6% preferred the ZONE variant. This option is preferred especially if future final restoration is to be bonded using resin cement. On the other hand, ZPC cement can chemically bond to the tooth structure and have minimal post–cementation sensitivity due to its large particle size. Therefore, some authors suggested its use as a long–term temporary cement [8, 11, 26].

The literature recommends the use of self–cure or dual–cure RC for the cementation of prefabricated glass fiber posts [27]. Light–cure RC is not recommended for the cementation of fiber posts due to the risk of incomplete polymerization [28]. The majority of the participants in this survey (57%) reported the use of RC for the cementation of fiber posts with 42.8% opting for dual–cure. This response suggests good understanding of the role of RC in bonding to tooth structure. Regarding custom–made cast posts and cores, the literature recommended the use of ZP due to its long–documented success and acceptable properties [7, 13, 26]. Its use has been reported by only 7.3% of the participants. The decline in ZP usage may be attributed to its high solubility, reduction of its use by academic institutions, and the emergence of more user–friendly cements that are easy to mix and apply. Similar findings were reported in the United Kingdom (UK), where the use of ZP has declined over time from 27 to 15% between 2008 and 2015 [29].

Conventional GIC use as a final cement for cast post and core has been recommended by some authors [11, 27]. In the current survey, 19.5% of the students/interns reported its use for this purpose. The high response for GIC may be related to its advantages such as chemical bonding, fluoride release, and resistance to acid dissolution. However, it is very sensitive to early moisture contamination in addition to the extended time needed to reach maturation (24–72 h) [13].

The use of RC for luting metallic posts had contradicting views but is gaining popularity because of the high tensile strength and ability to bond to dentin [4, 7, 26]. The use of light– or dual–cure RC is not recommended due to reduced bond strength and microhardness without light curing and uncertainty of complete polymerization before the tooth is subjected to stresses [7, 11]. About 30.2% reported the use of RC for custom–made cast posts and cores. Out of which, 21% used the chemical–cure, 69% used dual–cure, and 10% used light–cure versions. This might reflect the lack of students’ knowledge with regards to indications/contraindications and handling techniques of light–cure RC, which suggests the need for more clinical guidance.

The nature of lithium disilicate allows it to be acid etched in preparation for bonding to the tooth structure. For cases of full–coverage crowns with lithium disilicate, light– or dual–cure RC is suggested [7, 25]. This comes in agreement with the findings of the present survey; where 19.8% used dual–cure RC, and 12.6% used light–cured RC. The high selection of RC may be linked to guidance from supervising faculty, participant’s knowledge of restoration chemistry and the possibility to bond to the tooth structure, or the need to have a high degree of retention to compensate for defects in the tooth preparation.

Zirconia–based restorations being poly–crystalline in structure do not allow for etching of intaglio surface. However, it permits the use of conventional cements. Previous research recommended the use of RC, GIC, RMGIC, [7, 11] or ZP cement [13, 26]. The use of RC, particularly the light–cured version, was preferred by 27% of the participants. This may reflect a defect in the knowledge of the opaque nature of zirconia which may prevent or minimize the intensity of the light penetration. The findings of this study were slightly lower than those reported by Jum’ah et al. [29] who reported that half the dentists in UK use resin–based cements for cementation of zirconia crowns.

Concerning pressed porcelain laminate veneers, RCs are indicated for bonding [7, 13, 25, 26]. Out of the categories of RCs, a light–cured version is preferred due to color stability [7, 26]. The results of the current survey revealed that 34.6% selected RCs to bond veneers. Out of these, 43.3% only picked the light–cured version. The authors believe that this result might reflect the actual lack of information or inadequate clinical practice providing veneer treatments to patients where 45.7% of the participants reported no exposure to such cases.

Cement selection for CRIS restorations is determined based on implant location and orientation, occlusion, duration of retention required, ease of clean–up, and complexity of prosthesis among others [7, 26]. If all variables were favorable, and an extended period of retention is needed then a definitive dental cement such as GIC, [10, 26]. RMGIC, [4, 7, 26]. RC, [4, 7] or ZP [10, 13, 26] are recommended. If the restoration is to be temporarily retained and future retrieval is anticipated, ZOE may be used [13, 25]. In this survey, the majority of the respondents (55.6%) reported no experience with implant restoration, while 16.3% preferred RC, followed by GIC (8%), RMGIC (6.1%), ZO (6%), ZPC (4%), and finally ZP (3.7%). The findings were different from those described by Tarica et al. [30] who reported that the preference of restorative/prosthodontic department chairpersons was RMGIC followed by ZOE, GIC, RC, ZP and ZPC, while the implant program directors preferred RMGIC, ZOE and ZP. The difference in the reported results cannot be related to a single factor. However, Tarica et al. [30] study was surveying chairpersons and directors of implant and restorative programs at different US schools. Thus, more clinical experience and knowledge are expected to be reflected in their responses as compared to the results of this study.

Looking back at the study questions, (i) what were the sources of information used by dental students and interns to get information about luting cements? and (ii) were the clinical courses given to dental students during their undergraduate studies competent in providing the correct information to students regarding type, uses, precautions, and indications?, we can formulate the following answers: (i) it was confirmed that the main source of information regarding dental cements was the undergraduate studies which emphasize the importance of solid educational course to build–up students’ knowledge that would be used thereafter throughout their career. (ii) students satisfactorily considered the type of restorative material as the main factor when selecting the type of luting agent, and the results of student selection of cements per restoration indicated a significant lack of knowledge regarding indications of different types of cements which suggest the need of further improvement in the delivered didactic courses or clinical supervision.

### Strengths and limitations

The current study is the only study in KSA assessing the knowledge of dental students and interns towards dental cement uses, selection, and clinical applications. It provided information about the deficient sections in the curriculum of three top–ranked dental colleges that need enhancement in the upcoming years, which can be implemented in the curricula of the remaining dental colleges in the KSA. Also, the need to incorporate advanced treatment options such as laminate veneers, dental implant restorations and recent ceramic restorations to improve students’ skills and knowledge that is limited in these cases.The selection process of cement to be used for provisional or final restoration in academic institutions is very complex and multifactorial. The need to follow evidence–based procedures or the opinions of instructors in addition to the availability of selective types of dental cements in the clinics may have played a major role in the results of this study.

Among the limitations of this study is being cross–sectional in design so it cannot prove causality. Also, the study was restricted to the three major dental colleges in KSA.

Moreover, the survey was conducted among dental interns and dental students at different levels of education and clinical experience, and at multiple colleges with different curricula, teaching materials, and requirements, in addition to the variation in the availability and the types of dental cements used in the studied colleges which may have produced a wide range of answers and variations in responses. The teaching curricula provided the basic knowledge about luting cements and conventional prosthodontics procedures with possible lack of updates on newer generations of cements or restorative materials.

It is recommended to survey the undergraduate restorative and prosthodontic course directors to obtain their views and guidelines for the selection of cements in their respective courses and compare the results to those obtained from students. Also, dental students should be supervised in establishing their treatment plan and selecting the suitable material for each procedure with the lowest supervisor interference to improve their self–autonomy and decision–making skills.

## Conclusions

Within the limitations of the study, the following can be concluded:

1. Most of the respondents depended on undergraduate education as a source of information.
2. The choice of luting cement depends on the type and material of restoration, isolation technique, and the geometry of the tooth preparation.
3. Awareness towards management of gingival bleeding and restorations (laminate veneers or implant–supported restorations) is limited. However, this is not the case of provisional acrylic restorations, zirconia restorations and prefabricated glass fiber posts, since they are the most common procedures performed in the academic dental clinic.
4. Awareness towards the management of short prepared teeth and custom-made cast posts and cores is also limited.

## Electronic supplementary material

Below is the link to the electronic supplementary material.

Supplementary Material 1

## Data Availability

The datasets used and/or analyzed during the current study are available from the corresponding author on reasonable request.
