# Supplementary material for: Knowledge of Saudi dental students and interns towards luting cements and their applications in fixed prosthodontics
Source: BMC Oral Health. 2023 May 29;23:337. doi: 10.1186/s12903-023-03054-3 (PMC10228073; doi:10.1186/s12903-023-03054-3)
Supplement: Supplementary file 1 — Supplementary Material 1 [file 12903_2023_3054_MOESM1_ESM.docx]

**
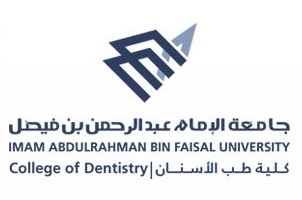
**

**Knowledge of Saudi dental students and interns towards luting cements and their applications in fixed prosthodontics**

Dear Participant,

We are conducting a study to assess practices related to the use of luting cement among dentists/ dental students in Saudi Arabia. Kindly respond to the following questions which will take about 5 minutes. Please notice that the questions assess your own practices and not your knowledge so feel free to share with us what you actually do. Your responses are confidential and will not be shared with anyone except the study team.

We appreciate your time.

1. **Personal and Professional Information**

Please select all that describes you of the following:

***I am a………***

| 1. Gender | - Male | | - Female | | |
| --- | --- | --- | --- | --- | --- |
| 1. Student | - No - Undergraduate (◌ 3^rd^ year/ ◌ 4^th^ year/ ◌ 5^th^ year/ ◌ 6^th^ year/ ◌ Intern) - Postgraduate | | | | |
| 1. Dentist | - No - Intern | | - General practitioner (GP) - Specialist/ Consultant | | |
| 1. The highest degree obtained | - None yet - BDS | | - Master - PhD/ DScD/ Equivalent | | |
| 1. Specialty | - None GP) - Endodontist - Operative dentist - Pediatric dentist - Other (non-clinical specialty) | | | - Prosthodontist - OMF surgeon - Periodontist - Orthodontist | |
| 1. Affiliation | - Academic sector - Governmental sector - Private sector | | | | |
| 1. Years of experience | - …...years | | | | |
| 1. School of undergraduate degree | - Saudi - North American - Asian | - Other Middle Eastern - European | | | |
| 1. School of postgraduate degree | - Saudi - North American - Asian | - Other Middle Eastern - European | | | |
| 1. How many patients do you manage/week? | | | | | ........patients |
| 1. What percentage of your patients requires the use of luting cements? | | | | | ……% |

1. **Luting Cement Use Practices**

Please select all that describes what you do or will do in each case (multiple selections are possible)

| 1. What are the sources you use to get information about luting cements? | 1. Undergraduate education 2. Continuing education 3. Internet | 1. Post graduate education 2. Journals & books 3. Colleagues |
| --- | --- | --- |
| 1. Which is the most important factor for you when selecting the type of luting cement? | 1. Type of restorative material 2. Accessibility for vision and isolation 3. Tooth preparation design and amount of retention needed 4. Availability of cement in clinic | |
| 1. What do you do when you encounter gingival bleeding during final cementation? | 1. Repeat temporary cementation and delay final cementation 2. Stop bleeding with ferric sulfate (Astringedent) and proceed with cementation 3. Stop bleeding with adrenaline and proceed with cementation 4. Stop bleeding with aluminium chloride (Gingi-aid) and proceed with cementation | |
| 1. What is the isolation technique you usually use for cementing laminate veneers? | 1. Split section rubber dam isolation 2. Individual tooth rubber dam isolation 3. Dry angle, cotton rolls and saliva ejector 4. Retraction cords | |

1. **Indicate the luting cement you usually use when you have the cases listed in column A:**
2. Zinc oxide eugenol cement
3. Zinc oxide non-eugenol cement
4. Zinc phosphate cement
5. Zinc polycarboxlate cement
6. Glass ionomer cement
7. Resin modified glass ionomer cement
8. Chemical cure resin cement
9. Dual cure resin cement
10. Light cure resin cement
11. I don’t do this type of restorations / or none of the above

| Cases (column A) | Cement I usually use for this case |
| --- | --- |
| 1. Short prepared tooth |  |
| 1. Provisional acrylic restoration |  |
| 1. Prefabricated glass fiber post |  |
| 1. Custom-made cast post & core |  |
| 1. Lithium disilicate (IPS e-max) restorations |  |
| 1. Zirconia based restorations |  |
| 1. Pressed porcelain laminate veneers |  |
| 1. Cement retained implant supported restorations |  |

Thank you
